# Effects of Cancer Cell-Derived Nanovesicle Vaccines Produced by the Oxidative Stress-Induced Expression of DAMP and Spontaneous Release/Filter Extrusion in the Interplay of Cancer Cells and Macrophages

**DOI:** 10.3390/biomedicines10081977

**Published:** 2022-08-15

**Authors:** Song-Hsien Lin, Guan-Ying Tsai, Meng-Jiy Wang, Szu-Yuan Chen

**Affiliations:** 1 Institute of Atomic and Molecular Sciences, Academia Sinica, Taipei City 106, Taiwan; 2 Department of Chemical Engineering, National Taiwan University of Science and Technology, Taipei City 106, Taiwan; 3 Department of Physics, National Central University, Taoyuan City 320, Taiwan

**Keywords:** cancer vaccine, nanovesicle, photodynamic therapy, oxidative stress, macrophage

## Abstract

Photodynamic therapy (PDT)-based cancer vaccines are shown to be more effective modalities for treating cancer in animal models compared to other methods used to generate cancer cell-derived vaccines. The higher efficacy seems to stem from the generation of cell membrane nanovesicles or fragments that carry both cancer cell-specific antigens and high surface content of damage-associated molecular pattern (DAMP) molecules induced by oxidative stress. To develop more effective cancer vaccines in this direction, we explored the generation of cancer vaccines by applying different sources of oxidative stress on cancer cell cultures followed by spontaneous release or filter extrusions to produce cancer cell-derived DAMP-expressing nanovesicles. Through an in-vitro test based on the co-culture of cancer cells and macrophages, it was found that the nanovesicle vaccines generated by H${}_{2}$O${}_{2}$ are as effective as those generated by PDT in diminishing cancer cell culture masses, providing a simpler way to manufacture vaccines. In addition, the nanovesicle vaccines produced by filter extrusion are as potent as those produced by spontaneous release, rendering a more stable way for vaccine production.

## 1. Introduction

Photodynamic therapy (PDT), which uses a photosensitizer in combination with light (of a suitable wavelength) to generate singlet oxygen that could damage tumor cells, is a promising method in cancer treatment [1,2,3,4,5,6]. However, the clinical application of PDT is limited by the short penetration depth of light. Moreover, it was found that the efficacy of PDT relies largely on its immunogenicity. Therefore, the production of a cancer vaccine by application of PDT to cultured tumor cells has been pursued as a promising route for cancer treatment [7], including the use of whole cells or their lysates [8,9,10] and the use of supernatants (secretions) [11]. Although the mechanisms of PDT-generated vaccines have not been completely resolved, researches suggest that there is a positive correlation between the amount of heat shock protein 70 (HSP70) expressed on the surface of PDT-treated cancer cells and the immunogenicity of the vaccine [12]. In addition, it was found that HSP70 translocates to the plasma membrane of cells after stress and is released into the extracellular environment in a membrane-associated form that can activate macrophages [13]. Moreover, a massive release of extracellular vesicles from cancer cells following photodynamic treatment or chemotherapy has been observed [14]. All of these together indicate that the cancer vaccines made from the supernatants of PDT-treated cancer cells could be nanovesicles carrying cancer cell surface antigens and HSP70. They can be engulfed by macrophages and dendritic cells, inducing the release of cytokines and the presentation of cancer antigens to T cells. In fact, HSP70 has been studied as an adjuvant for cancer immunotherapy [15]. It has been found that HSP70 can activate dendritic cells partly through toll-like receptors, activate natural killer cells, increase the presentation of antigens to effector cells, and augment T-cell and humoral immune responses against their associated antigens [15]. The fact that PDT-generated vaccines are more effective than vaccines produced by other modalities supports the importance of the co-delivery of HSP70 and/or other damage-associated molecular pattern (DAMP) molecules with antigens [3,11]. This is consistent with research on using exosomes to co-deliver tumor antigens and immunostimulatory factors for cancer immunotherapy [16,17].

It has been shown that cancer vaccines produced by a collection of nanovesicles spontaneously released from PDT treatment of excised cancer cells are effective in inhibiting tumor growth in animal tests [11]. It was also found that T-cell immunity mediated by dendritic cells plays an important role in the efficacy of such vaccines [9,11,18]. On the other hand, past studies have also shown that the interactions between cancer cells and tumor-associated macrophages (TAMs) in tumors play a critical role in the effectiveness of cancer immunotherapy [19,20,21,22]. In a malignant tumor, TAMs are co-opted by the tumor cells and convert into pro-tumor M2 phenotypes instead of anti-tumor M1 phenotypes. The repolarization of M2 to M1 macrophages has become an important strategy for cancer treatment [23]. In addition, some studies pointed out that the leaky neovasculature in tumors allows nanoparticle-packaged drugs to enter and accumulate in tumors, rendering the effect of targeted therapy [24,25]. The targeting effect is even stronger with cell-derived nanovesicles [26,27]. Based on these findings, it seems that vaccines made of nanovesicles generated by PDT may also directly enter tumors through blood circulation and affect the interplay of cancer cells and TAMs, thereby contributing to the efficacy of the vaccines. Therefore, in this work, we used a co-culture of cancer cells and macrophages to test the effect of adding a vaccine to simulate and clarify the role of the PDT nanovesicle vaccine in this regard.

Furthermore, the above-mentioned PDT nanovesicle vaccine is produced by exerting oxidative stress on cancer cells by PDT to induce the expression of DAMP molecules, such as HSP70 and calreticulin on cell membranes, followed by the spontaneous release of nanovesicles with these DAMPs on the surfaces. Therefore, in this work, we also explored the schemes of adding H${}_{2}$O${}_{2}$ directly in cancer cell cultures to generate oxidative stress and used mechanical extrusion through a filter to generate nanovesicles, by using the co-cultures of cancer cells and macrophages to compare the efficacies of vaccines produced with these different methods. In particular, past studies showed that the most effective light dose for inducing the spontaneous release of nanovesicles from PDT-treated cancer cells is lower than the dose needed for inducing immunogenic apoptosis that can generate DAMP [12,14]. In addition, the timing of the release of nanovesicles seems earlier than the time needed for maximal DAMP expression [12,14]. Therefore, it might be difficult to achieve the highest DAMP expression and the highest nanovesicle number at the same time with that method. By using the optimal dose for maximizing DAMP expression and using filter extrusion at the optimal timing (recovery time) to produce nanovesicles, such a trade-off could be overcome to achieve a higher vaccine efficacy. In addition, previous studies have shown that nanovesicles spontaneously released by cancer cells (exosomes) may have the adverse effect of promoting cancer cell proliferation and metastasis [28,29]. Since the pathway of generating nanovesicles by filter extrusion is inherently different from that of spontaneous release, i.e., through direct outward budding (similar to that for ectosomes) [30,31] rather than through an endosomal pathway (double invagination of the plasma membrane, such as that for exosomes) [14], it is necessary to compare the direct effects of nanovesicles produced by these two different methods on cancer cells to reveal its impact on the overall immune effect.

In this study, first the surface HSP70 expression and calreticulin expression of mouse Lewis lung carcinoma (LLC) cells treated by different oxidative stresses (PDT and H${}_{2}$O${}_{2}$) were characterized. Next, nanovesicles produced from the treated LLC cells by spontaneous release and filter extrusion, respectively, were collected through ultracentrifugation as vaccines. Then, the effectiveness of these vaccines was assessed by three in-vitro tests. The first test was to measure the NO secretions of mouse macrophages treated with various vaccines, because NO is one of the major hallmarks of the M1 phenotype of macrophages [32] and one of the major cytotoxic factors of macrophages for killing cancer cells [33]. The second test was to examine the viability of LLC cells co-cultured with macrophages in the presence of various vaccines to assess the efficacy of these vaccines in killing LLC cells in the condition simulating tumor microenvironment. The last test was to measure the direct effects of the vaccines on LLC cells without the presence of macrophages in order to clarify the action pathways of the vaccines in the co-cultures of LLC cells and macrophages.

## 2. Methods

### 2.1. Cell Culture

Mouse Lewis lung carcinoma (LLC) cells LL/2 of the C57BL strain and mouse macrophages J774A.1 of the BALB/c strain were purchased from the Bioresource Collection and Research Center, Taiwan. Cells were maintained in Dulbecco’s Minimum Essential Medium (DMEM) supplemented with 10% fetal bovine serum (FBS) (GE Healthcare Life Sciences, Piscataway, NJ, USA). Cell cultures were incubated in a cell incubator with a humidified atmosphere of 5% CO${}_{2}$ and 37 ${}^{\circ }$C and sub-cultured every two days.

### 2.2. Production of Various Oxidative Stresses

For H${}_{2}$O${}_{2}$ treatment, LLC cells were incubated in DMEM with various concentrations of H${}_{2}$O${}_{2}$ and then placed in the cell incubator for 24 h. For photodynamic therapy (PDT) treatment, LLC cells were first incubated in DMEM containing various concentrations of verteporfin (Sigma-Aldrich, St. Louis, MO, USA) for 1 h and then irradiated with a laser beam of a 690-nm wavelength for various accumulated fluences. After that, the medium was replaced by a medium without verteporfin and the treated cells were placed in the cell incubator for various durations (recovery times).

### 2.3. Collection of Spontaneously-Released Nanovesicles and Production of Nanovesicles via Filter Extrusion as the Vaccine

For collecting nanovesicles released spontaneously from LLC cells, i.e., extracellular vesicles, the supernatant from the treated cell culture was collected after various recovery times and centrifuged at 90× *g* for 10 min. Then, the supernatant was collected and subjected to centrifugation at 350× *g* for 10 min to isolate cell debris in a pellet. After that, the supernatant was collected and subjected to ultra-centrifugation at 100,000× *g* at 4 ${}^{\circ }$C for 2 h to isolate the nanovesicles in a pellet. The nanovesicles were then resuspended in DMEM, referred to as the vaccine. For the production of nanovesicles by filter extrusion, the treated cells were collected after various recovery times and then loaded into a pneumatic filter extruder operating at 80 psi air pressure with a polycarbonate filter (Whatman) with a 0.4-μm pore diameter [30,31,34]. The extrusion solution was collected and then subjected to the same serial centrifugation process (350× *g* and 100,000× *g*) as described above to produce the vaccine [34].

### 2.4. Characterization of Cell Surface Expression of HSP70 and Calreticulin

To measure the surface HSP70 expressions of LLC cells, the treated cells were collected after various recovery times and resuspended in 0.5 mL of PBS. A total of 2 μL of anti-HSP70 antibody conjugated Alexa Fluor 647 (Bioss) was then added and the sample was measured with a flow cytometer (BD FACSCalibur). To measure the surface calreticulin expressions of LLC cells, the treated cells were collected after various recovery times and resuspended in 0.5 mL of PBS. Then, 2 μL of anti-calreticulin antibody conjugated fluorescein isothiocyanate (FITC) (Novus) was added and the sample was measured with the flow cytometer.

### 2.5. Characterization of Nanovesicles

Nanovesicle number and size distributions were measured using the nanoparticle tracking analysis (NTA) (NanoSight NS300). The sample was diluted (10×) in PBS and a 300 μL aliquot was loaded into the observation chamber and recorded for 60 s. The video was analyzed with NanoSight software. For direct observation of nanovesicles, a 300 μL aliquot of the sample was dropped on a mica plate, fixed with 4% paraformaldehyde, air-dried, and then observed with atomic force microscopy (AFM) [35].

### 2.6. Measurement of NO Secretion from Macrophages and Imaging of NO Expression Inside Macrophages

Macrophages were incubated in a 96-well plate (Nest) until over 90% confluency. Then the medium was removed, 200 μL of the vaccine in DMEM was added, and the sample was incubated in the cell incubator for 24 h to prime (activate, train) the macrophages. The cell culture supernatant was then collected, mixed with an equal volume of Griess reagent (1% sulfanilamide and 0.1% naphthylethylenediamine in 5% phosphoric acid), and incubated at room temperature for 30 min. The absorbance at 560 nm was recorded using a microplate reader (Promega GloMax Discover). For taking fluorescence images of NO expression inside the macrophages, macrophages were incubated in an 8-well glass slide (Cellvis) for 2 h. Then the medium was removed, 200 μL of 100 ng/mL of LPS in DMEM was added, and the sample was incubated in the cell incubator for 24 h to prime the macrophages. The primed macrophages were stained with 4-amino-5-methylamino-2’,7’-difluorofluorescein diacetate (DAF-FM Diacetate), and then fluorescence images of NO distribution were taken with a confocal microscope (Leica SP-8).

### 2.7. Characterization of the Mortality of Cancer Cells Co-Cultured with Macrophages and/or Vaccine

For characterization of the effect of the vaccine on LLC cells co-cultured with macrophages, LLC cells and macrophages were stained with CellTrace Far Red and CellTrace CFSE (Thermo Fisher Scientific, Waltham, MA, USA), respectively. The cells were centrifuged at 90× *g* for 5 min and the cell pellet was resuspended in PBS at a concentration of 10${}^{6}$ cells/mL. Moreover, 1 μL of Cell Trace solution was then added to 1 mL of cell suspension and then the cells were incubated in the cell incubator (light-tight) for 20 min. After that, the cells were centrifuged at 90× *g* for 5 min, resuspended in DMEM with 10% FBS at a concentration of 10${}^{6}$ cells/mL for 5 min, centrifuged again to remove any remaining free dye, and then resuspended in DMEM. The stained LLC cells and macrophages were then co-cultured at a ratio of 1:2 in 1 mL of vaccine-loaded DMEM (without FBS) supplemented with antibiotic (1 *v*/*v*% penicillin–streptomycin–neomycin solution stabilized from Sigma-Aldrich) in a non-passivated 35-mm dish for 48 h. After that, all cells were collected and measured with the flow cytometer to obtain the number of intact LLC cells. For observation of the effect of the vaccine on LLC cells directly, the same protocol as the above was used except that macrophages were not added.

### 2.8. Statistical Analysis

All data in bar diagrams are presented as mean ± standard deviations over three or four replicates as indicated in the figure captions. Statistical significance was evaluated by one-way ANOVA with Tukey’s test to obtain a *p* value. A value of *p* < 0.05 was regarded as statistically significant.

## 3. Results

### 3.1. HSP70 Expression of LLC Cells Treated with Various Sources of Oxidative Stress

**Figure 1:**
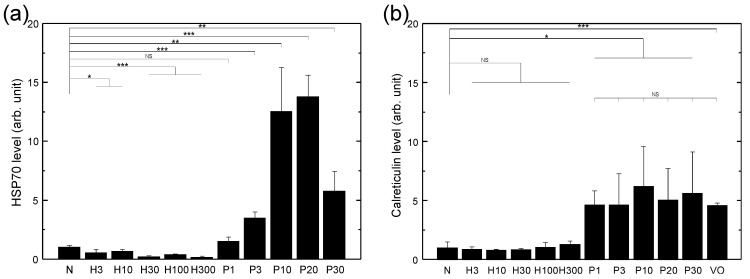
Surface HSP70 (**a**) and calreticulin (**b**) expressions of LLC cells treated with various sources of oxidative stress after 24 h of recovery time. The HSP70 and calreticulin levels were normalized to that of the respective control group (N). N: medium only; H3, H10, H30, H100, H300: treated with 3, 10, 30, 100, and 300 μM H${}_{2}$O${}_{2}$ respectively; P1, P3, P10, P20, P30: treated with PDT using 4 μg/mL of verteporfin and a light fluence of 1, 3, 10, 20, and 30 J/cm${}^{2}$, respectively; VO: verteporfin only (without light). Data are expressed by mean ± SD (N = 4), NS: no significant difference, *: *p*-value < 0.05, **: *p*-value < 0.01, and ***: *p*-value < 0.001.

**Figure 2:**
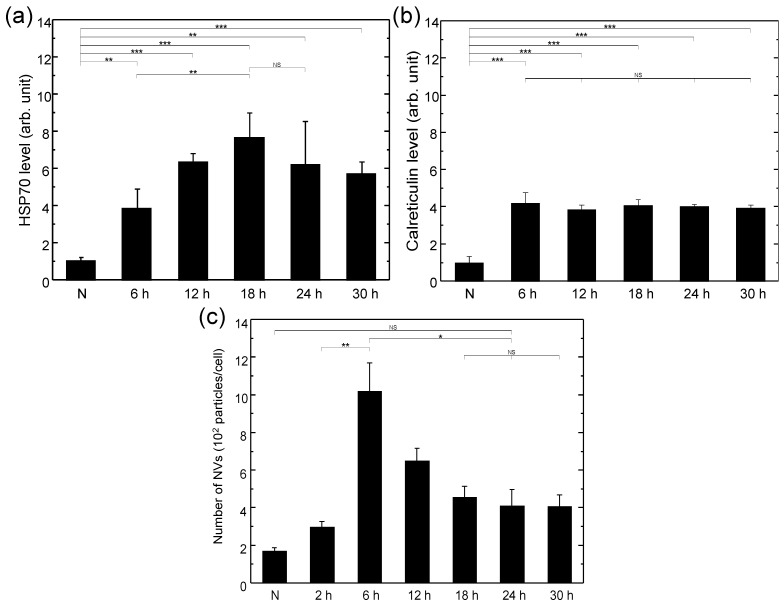
(**a**) Surface HSP70 expression of LLC cells as a function of recovery time after PDT treatment with 4 μg/mL of verteporfin and 10 J/cm${}^{2}$ fluence. The HSP70 level was normalized to that of the control group (N). N: without PDT treatment. (**b**) Surface calreticulin expression of LLC cells as a function of recovery time after PDT treatment with 4 μg/mL of verteporfin and 20 J/cm${}^{2}$ fluence. The calreticulin level was normalized to that of the control group (N). N: without PDT treatment. (**c**) The number of spontaneously released nanovesicles as a function of time after PDT treatment. N: number of nanovesicles spontaneously released from LLC cells in a time span of 24 h without PDT treatment. Data are expressed by mean ± SD ((**a**) N = 4, (**b**) N = 4, (**c**) N = 3), NS: no significant difference, *: *p*-value < 0.05, **: *p*-value < 0.01, and ***: *p*-value < 0.001.

In this work, the dependence of the HSP70 expression level on the verteporfin concentration with the same light fluence was tested first. It was found that the HSP70 expression level produced with a verteporfin concentration of 40 μg/mL was not significantly higher than that with 4 μg/mL. Therefore, a verteporfin concentration of 4 μg/mL was chosen for all subsequent experiments to render the highest effect while avoiding cytotoxicity induced directly by the photosensitizer alone. Figure 1 shows the surface HSP70 and calreticulin expressions of LLC cells treated with PDT and H${}_{2}$O${}_{2}$ of various doses, respectively, after 24 h of recovery time. It was observed that PDT was very effective at inducing cell surface expression of both HSP70 and calreticulin, and the optimal light dose of PDT was around 10–20 J/cm${}^{2}$. It was also observed that HSP70 and calreticulin were both expressed on LLC cells without stress (group N), and upon stress by PDT with a light dose of 20 J/cm${}^{2}$, the surface expression of HSP70 increased by *ca.* 14 times and that of calreticulin increased by *ca.* 5 times after 24 h. In contrast, adding H${}_{2}$O${}_{2}$ slightly decreased the HSP70 expression and did not affect calreticulin expression. The strong dependence of HSP70 expression on the light dose indicated that the upregulated HSP70 expression with respect to the control (group N) was due to a photodynamic process. On the contrary, it was found that the upregulated calreticulin expression showed no light dose dependence and was about the same as that without light irradiation (group VO). This indicated that verteporfin by itself could upregulate the surface calreticulin expression of LLC cells and adding light irradiation could not raise it any further. The surface HSP70 and calreticulin expressions of LLC cells as functions of recovery time after PDT treatment are shown in Figure 2a,b respectively. It was found that the surface HSP70 expression increased with time at first, reaching the maximum at around 18–24 h, and then decreased gradually. In comparison, the expression of calreticulin rose even more rapidly, reached saturation within 6 h, and maintained at the same level over 30 h. Therefore, a recovery time of 24 h was used in the subsequent experiments for generating vaccines through both spontaneous release and filer extrusion to render a higher overall DAMP level on vaccine nanovesicles.

### 3.2. Nanovesicles Produced by Spontaneous Release and Filter Extrusion

**Figure 3:**
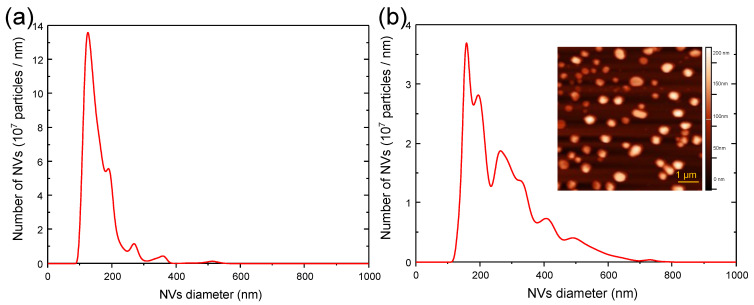
Size distributions of nanovesicles (NVs) produced by (**a**) spontaneous release and (**b**) filter extrusion, respectively, following PDT treatment of LLC cells with 4 μg/mL of verteporfin, 20 J/cm${}^{2}$ light fluence, and 24 h of recovery time, measured with NTA. LLC cell number: 2.2 × 10${}^{7}$. Inset of (**b**): morphology of nanovesicles produced by filter extrusion and measured with AFM.

Figure 3 shows the size distributions of nanovesicles produced by spontaneous release and filter extrusion, respectively, following PDT treatment. The diameters of the nanovesicles produced by spontaneous release (i.e., exosomes/extracellular vesicles) ranged from 100 to 350 nm, while that produced by filter extrusion with 0.4 μm filter ranged from 100 to 650 nm. The morphology of the nanovesicles was visualized and the size was confirmed with AFM, as shown in the inset of Figure 3b. It was found that the number of nanovesicles produced by filter extrusion was around 400 per cell and was roughly the same for various treatments and recovery times. In contrast, it was observed that the number of nanovesicles produced by spontaneous release varied with different treatment conditions substantially. In addition, as can be seen in Figure 2c, the number of spontaneously released nanovesicles reached the maximum within 6 h and then decreased to and stabilized at about half of the maximum after 18 h. The number of nanovesicles spontaneously released from LLC cells in a time span of 24 h without any externally applied stress is plotted as group N for comparison. The results showed that the LLC cells released nanovesicles massively following PDT treatment, consistent with the findings of previous works [14].

### 3.3. NO Secretion and Morphology Change of Macrophages Stimulated by Various Vaccines

**Figure 4:**
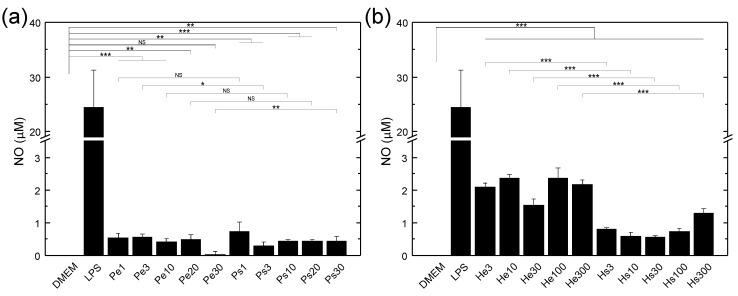
NO secretion of macrophages measured at 24 h after being treated with various vaccines produced by PDT (**a**) or H${}_{2}$O${}_{2}$ (**b**) through spontaneous release or filter extrusion. DMEM: medium only; LPS: with 100 ng/ml lipopolysaccharide (LPS); Pe1, Pe3, Pe10, Pe20, Pe30: vaccines prepared using PDT with 4 μg/mL of verteporfin and a light fluence of 1, 3, 10, 20, and 30 J/cm${}^{2}$, respectively, followed by filter extrusion after 24 h of recovery time; Ps1, Ps3, Ps10, Ps20, Ps30: vaccines prepared using PDT with 4 μg/mL of verteporfin and a light fluence of 1, 3, 10, 20, and 30 J/cm${}^{2}$, respectively, with the supernatant collected after 24 h of recovery time; He3, He10, He30, He100, and He300: vaccines prepared using 3, 10, 30, 100, and 300 μM H${}_{2}$O${}_{2}$, respectively, followed by filter extrusion after 24 h of recovery time; Hs3, Hs10, Hs30, Hs100, and Hs300: vaccines prepared using 3, 10, 30, 100, and 300 μM H${}_{2}$O${}_{2}$, respectively, with the supernatant collected after 24 h of recovery time. For the vaccine groups, each well contained 1 × 10${}^{5}$ macrophages, and the nanovesicle vaccines made from 5.2 × 10${}^{6}$ LLC cells. Data are expressed by mean ± SD (N = 4), NS: no significant difference, *: *p*-value < 0.05, **: *p*-value < 0.01, and ***: *p*-value < 0.001.

Figure 4 shows the amount of NO secretion of macrophages treated with vaccines produced by using PDT or H${}_{2}$O${}_{2}$ through filter extrusion or spontaneous release. In all experiments, the negative control with only the medium was used to indicate the background level of NO in a culture of macrophages without stimulants, and the positive control with LPS served as a scale for evaluating the effectiveness of macrophage activation. As can be seen, all vaccine groups were effective at activating macrophages, with NO levels much higher than without adding the vaccine (DMEM group, which was zero or even negative after subtracting the background). Moreover, the vaccine produced by filter extrusion was more effective than that produced by spontaneous release regardless of the dose used when H${}_{2}$O${}_{2}$ was used, while the difference was mostly insignificant when PDT was used. In addition, the vaccine generated by H${}_{2}$O${}_{2}$ was in general more effective than that by PDT for both the filter extrusion and spontaneous release.

**Figure 5:**
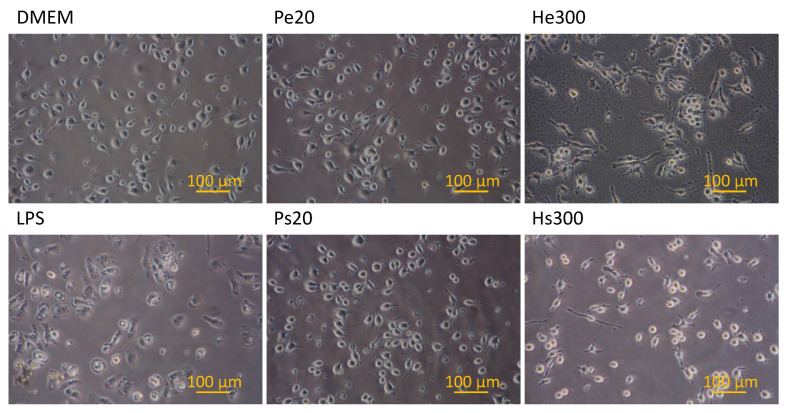
Morphology of macrophages after incubation with various vaccines for 24 h. DMEM: medium only; LPS: with 100 ng/mL lipopolysaccharide; Pe20: vaccine prepared using PDT with 4 μg/mL of verteporfin at 20 J/cm${}^{2}$ fluence followed by filter extrusion after 24 h of recovery time; Ps20: vaccine prepared using PDT with 4 μg/mL of verteporfin at 20 J/cm${}^{2}$ fluence, with the supernatant collected after 24 h of recovery time; He300: vaccine prepared using 300 μM H${}_{2}$O${}_{2}$ followed by filter extrusion after 24 h of recovery time; Hs300: vaccine prepared using 300 μM H${}_{2}$O${}_{2}$, with the supernatant collected after 24 h of recovery time. Each dish contained 3 × 10${}^{5}$ macrophages, and the nanovesicle vaccine made from 5.2 × 10${}^{6}$ LLC cells.

**Figure 6:**
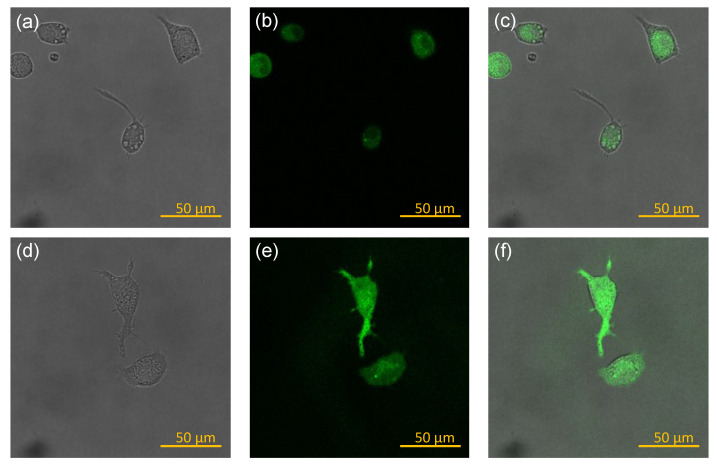
NO staining of macrophages at 24 h after being treated with 100 ng/mL of LPS: (**a**,**d**) optical microscopy images; (**b**,**e**): confocal microscopy images; (**c**,**f**): superposition of the two.

Figure 5 shows the morphology of macrophages after being incubated with various vaccines for 24 h. Without stimulation, a small percentage of macrophages displayed filopodia and/or cytoplasmic vacuoles (group DMEM). Upon stimulation by LPS (group LPS), almost all macrophages exhibited cytoplasmic vacuoles and both the number and size of vacuoles increased substantially, but very few macrophages displayed filopodia. Upon stimulation by the various vaccines, both the percentages of macrophages expressing filopodia and the length of filopodia increased compared to non-stimulated macrophages (group DMEM). Furthermore, the expression of filopodia was stronger in the case using the He300 vaccine compared to those using the other vaccines, which was in positive correlation with the NO secretion shown in Figure 4. The particles observed in the image of He300 could be attributed to plasma membrane-derived particles from macrophages, the presence of which was consistent with the long filopodia expressed in that case [36]. NO staining of the macrophages, as shown in Figure 6, revealed that all the macrophages stimulated by LPS expressed NO in the cytoplasm, indicating that they all differentiated into M1 phenotype macrophages after the stimulation but with varying degrees of activation (varying NO amounts).

### 3.4. Mortality of LLC Cells Co-Cultured with Macrophages and/or Treated with Various Vaccines

**Figure 7:**
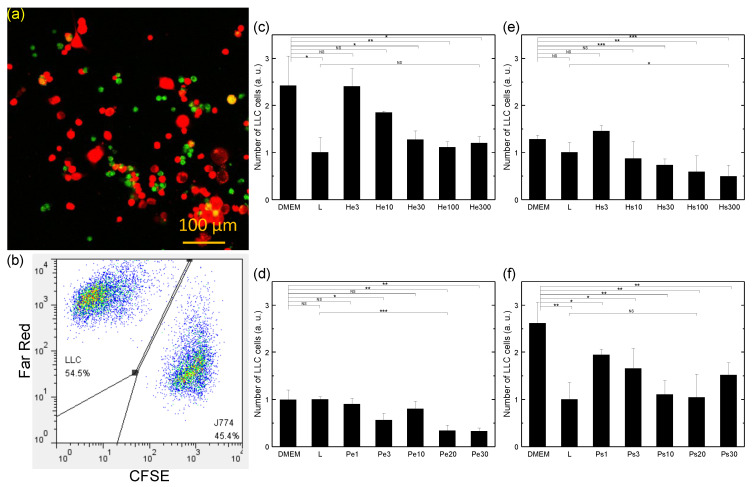
Numbers of viable LLC cells after co-culturing with macrophages and treated with various vaccines for 48 h. (**a**) Fluorescence image of the co-cultured LLC cells (red) and macrophages (green) at 48 h after plating without any vaccine. (**b**) Flow cytometry dot graph of the co-cultured LLC cells (Far Red) and macrophages (CFSE) at 48 h after plating without adding the vaccine. (**c**) Comparison of H${}_{2}$O${}_{2}$-extrusion vaccines with various doses. (**d**) Comparison of PDT-extrusion vaccines with various doses. (**e**) Comparison of H${}_{2}$O${}_{2}$-supernatant vaccines with various doses. (**f**) Comparison of PDT-supernatant vaccines with various doses. The initial ratio of the number of LLC cells and macrophages was 1:2. DMEM: without the vaccine; L: without macrophages and vaccines; He3, He10, He30, He100, and He300: vaccines prepared using 3, 10, 30, 100, and 300 μM H${}_{2}$O${}_{2}$, respectively, followed by filter extrusion after 24 h of recovery time; Hs3, Hs10, Hs30, Hs100, and Hs300: vaccines prepared using 3, 10, 30, 100, and 300 μM H${}_{2}$O${}_{2}$, respectively, with the supernatant collected after 24 h of recovery time; Pe1, Pe3, Pe10, Pe20, and Pe30: vaccines prepared using PDT with 4 μg/mL of verteporfin and a light fluence of 1, 3, 10, 20, and 30 J/cm${}^{2}$, respectively, followed by filter extrusion after 24 h of recovery time; Ps1, Ps3, Ps10, Ps20, and Ps30: vaccines prepared using PDT with 4 μg/mL of verteporfin and a light fluence of 1, 3, 10, 20, and 30 J/cm${}^{2}$, respectively, with the supernatant collected after 24 h of recovery time. Each dish contained 1.5 × 10${}^{5}$ LLC cells, 3 × 10${}^{5}$ macrophages (if added), and the nanovesicle vaccine made from 5.2 × 10${}^{6}$ LLC cells. Data are expressed by mean ± SD (N = 4), NS: no significant difference, *: *p*-value < 0.05, **: *p*-value < 0.01, and ***: *p*-value < 0.001.

The cytotoxicity of macrophages towards co-cultured LLC cells in the presence of various vaccines was studied in vitro. Figure 7a,b show the fluorescence image and flow cytometry dot graph of the co-cultured LLC cells and macrophages at 48 h after plating without adding the vaccine. The dot plots show two distinctive groups of cells, permitting unambiguous determination of the numbers of the two types of cells by assigning the cells with a stronger Far Red signal to LLC cells and the cells with stronger CFSE signals to macrophages. Cells were determined viable (intact) if their forward scattering and side scattering signal intensities fell in the range of those for the cells before co-culture. The number of viable LLC cells in each co-culture was obtained by dividing the number of viable LLC cells counted in the dot graph by the aspiration volume (product of the fixed data acquisition time and the fixed sample flow rate) and then multiplying by the total volume of the sample. Figure 7c–f show the numbers of viable LLC cells measured at 48 h after co-culture of the LLC cells and macrophages in the presence of vaccines made by H${}_{2}$O${}_{2}$-extrusion (c), PDT-extrusion (d), H${}_{2}$O${}_{2}$-supernatant (e), and PDT-supernatant (f), respectively with various doses. Comparing the numbers of viable LLC cells in the cases of DMEM and L in all four experiments, it was found that without the vaccine, the number of LLC cells could be more than doubled after 48 h in the presence of macrophages (DMEM vs. L in Figure 7c,f). This indicated that the co-culturing of macrophages with cancer cells could often facilitate the proliferation of cancer cells, as is commonly known for the effects of tumor-associated macrophages. When the vaccine was added to the culture, it was clearly seen that the number of viable LLC cells in the co-culture of LLC cells and macrophages was generally lower than that without the vaccine (DMEM) and the number decreased with the increased oxidative stress dose (PDT light fluence and H${}_{2}$O${}_{2}$ concentration) until reaching an optimal dose (20 J/cm${}^{2}$ for PDT light fluence and 300 μM for H${}_{2}$O${}_{2}$ concentration) regardless of using the extrusion or supernatant. Moreover, the number of viable LLC cells could be about the same as that of LLC cells incubated alone (He300 vs. L in Figure 7c and Ps20 vs. L in Figure 7f) or even much lower than that of LLC cells incubated alone (Pe20 vs. L in Figure 7d and Hs300 vs. L in Figure 7e). This indicated that the presence of the nanovesicle vaccines in the co-culture of cancer cells and macrophages could overturn or override the effects of macrophages in promoting LLC cell proliferation to suppress the growth of the cancer cell culture or even substantially reduce the cancer cell culture mass. The highest efficacy of killing LLC cells was achieved with the vaccine prepared using PDT with 4 μg/mL of verteporfin at 20 J/cm${}^{2}$ light fluence followed by filter extrusion (Pe20, Figure 7d), resulting in a dramatic shrinkage of the LLC cell culture mass to 34% of that with LLC cells alone (L).

**Figure 8:**
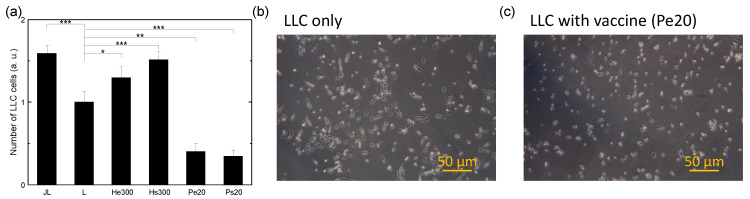
Numbers of viable LLC cells after being treated with various vaccines for 48 h in the absence of macrophages. (**a**) Comparison of various vaccines. JL: without the vaccine but with the macrophage; L: without the vaccine; He300: vaccine prepared using 300 μM H${}_{2}$O${}_{2}$ followed by filter extrusion after 24 h of recovery time; Hs300: vaccine prepared using 300 μM H${}_{2}$O${}_{2}$ with the supernatant collected after 24 h of recovery time; Pe20: vaccine prepared using PDT with 4 μg/mL of verteporfin and a light fluence of 20 J/cm${}^{2}$ followed by filter extrusion after 24 h of recovery time; Ps20: vaccine prepared using PDT with 4 μg/mL of verteporfin and a light fluence of 20 J/cm${}^{2}$, with the supernatant collected after 24 h of recovery time. Data are expressed by mean ± SD (N = 4), NS: no significant difference, *: *p*-value < 0.05, **: *p*-value < 0.01, and ***: *p*-value < 0.001. (**b**) Morphology of LLC cells without treatment by the vaccine. (**c**) Morphology of LLC cells after being treated by the vaccine prepared using PDT with 4 μg/mL of verteporfin and a light fluence of 20 J/cm${}^{2}$ followed by filter extrusion after 24 h of recovery time. Each dish contained 1.5 × 10${}^{5}$ LLC cells, 3 × 10${}^{5}$ macrophages (if added), and the nanovesicle vaccine made from 5.2 × 10${}^{6}$ LLC cells.

To clarify whether the cytotoxicity of the vaccines toward LLC cells was mediated by macrophages or acted directly on LLC cells, the numbers of viable LLC cells after being treated with various vaccines for 48 h in the absence of macrophages were measured, as shown in Figure 8. It was found that the vaccines made with PDT could directly kill LLC cells regardless of using the extrusion or supernatant, lowering the number of viable LLC cells to 40% of that without the vaccine with the Pe20 vaccine and 34% with the Ps20 vaccine. The morphologies of the LLC cells for the cases without the vaccine and with the Pe20 vaccine, as shown in Figure 8b,c respectively, showed that the percentages of the LLC cells displaying elongated shapes were significantly reduced, consistent with the drop in the number of viable LLC cells observed with flow cytometry. On the contrary, the vaccines made by H${}_{2}$O${}_{2}$ could increase viable LLC cell numbers regardless of using the extrusion or supernatant, raising the number of viable LLC cells by 29% with the He300 vaccine and 51% with the Hs300 vaccine. This experiment was repeated several times. The increase in the number of viable LLC cells with vaccines made by H${}_{2}$O${}_{2}$ and the decrease with vaccines made by PDT were reproducible, but the degree of the drop with vaccines made by PDT varied substantially among the experiments.

## 4. Discussion

It is well-known that HSP70 expression on the plasma membrane is a signature of cancer cells. It occurs on primary tumor cells and distant metastases, but not on corresponding normal cells and tissues [37,38]. In this work, it was observed that HSP70 and calreticulin were expressed on LLC cells without stress (group N in Figure 1), consistent with the past studies. It was also found that the surface expression of HSP70 increased by *ca.* 14 times after 24 h with PDT of the optimal dose (Figure 1a), similar to the *ca.* 10-fold increase of the HSP70 surface expression after 18 h, observed in a previous report [12]. Distinctively, stressed by H${}_{2}$O${}_{2}$, the surface expression of HSP70 dropped and that of calreticulin increased at most by just a fraction (Figure 1). This is also consistent with a previous report [39], which showed an increase of merely *ca.* 40% in the HSP70 surface expression and *ca.* 80% in the calreticulin surface expression stressed by 100 μM H${}_{2}$O${}_{2}$ after 24 h.

In the co-culture of LLC cells and macrophages simulating the tumor microenvironment [40,41], it was seen that the nanovesicle vaccines could reduce the number of viable LLC cells and the efficacy was higher with increasing doses (until reaching an optimal value) no matter which oxidative stress was used and which way of nanovesicle production was adopted (Figure 7). In addition, the macrophages displayed varying degrees of ability in promoting LLC cell proliferation in different rounds of experiments: more than double in Figure 7c,f, by a fraction in Figure 7e, and no effect in Figure 7d. Therefore, overall, it seems that the vaccines could completely inhibit the growth of cancer cell culture masses when the macrophages have strong promoting effects on LLC cell proliferation and could reduce cancer cell culture masses substantially when the macrophages have weak promoting effects. This means that the nanovesicle vaccines made with PDT or H${}_{2}$O${}_{2}$ and extrusion or spontaneous release (accumulated and retained in a tumor) could all aid T-cell immunity against cancer. However, the relative overall efficacies of these nanovesicle vaccines in treating cancer need to be explored through animal testing, because these different vaccines may have different potencies in activating dendritic cells and T cells. The effectiveness of vaccines in the form of nanovesicles carrying both DAMP molecules and antigens in activating macrophages could be attributed to the better uptake and antigen processing of particulate vaccines by macrophages [42].

Combining the results of the cytotoxic effects of vaccines on LLC cells in the presence of macrophages (Figure 7) and that without macrophages (Figure 8), it seems that, in the co-culture of LLC cells and macrophages, the vaccines made by H${}_{2}$O${}_{2}$ kill LLC cells predominantly through the activation of the cytotoxicity of macrophages toward LLC cells, since the vaccine nanovesicles by themselves could promote LLC cell proliferation to some extent upon uptake by LLC cells. In contrast, the vaccines made by PDT kill LLC cells through both uptake of vaccine nanovesicles by LLC cells and activation of macrophages, since they show the ability to directly reduce LLC cell numbers in varying degrees. The experiments of NO secretion of macrophages stimulated by various vaccines (Figure 4) show that the concentration of NO secreted from macrophages with vaccines made using H${}_{2}$O${}_{2}$ is much higher than that with vaccines made using PDT. This is consistent with the higher cytotoxicity of macrophages toward LLC cells with vaccines made by H${}_{2}$O${}_{2}$. The much lower (but still significant) NO level secreted from macrophages stimulated by vaccines made with PDT indicates that either the macrophages are less activated or they secret other types of cytotoxic factors more prominently. The induction of proliferation of LLC cells by the nanovesicle vaccines made by H${}_{2}$O${}_{2}$ is not a surprise, because it is well known that exosomes produced from cancer cells may enhance tumor growth and metastasis [14]. On the other hand, the effectiveness of the vaccines made by PDT in directly killing LLC cells might be ascribed to the very high HSP70 and calreticulin expression levels on the vaccine nanovesicles (Figure 1), which might lead to apoptosis of the LLC cells upon uptake. Compared to PDT, with vaccines made by H${}_{2}$O${}_{2}$, the NO secretion level from macrophages is much higher even though the HSP70 and calreticulin expression levels on the vaccine nanovesicles are much lower. One possible explanation is that the DAMP molecules induced by H${}_{2}$O${}_{2}$ are of different types from those induced by PDT (HSP70 and calreticulin) as a result of damaging different parts of cells (endoplasmic reticulum with PDT using verteporfin vs. plasma membrane with H${}_{2}$O${}_{2}$) [3], which may render the vaccine made by H${}_{2}$O${}_{2}$ more effective in activating macrophages but less effective in directly inducing LLC cell apoptosis. One possibility of the DAMP molecules expressed on cancer cell surfaces caused by H${}_{2}$O${}_{2}$ is the oxidation-damaged molecular complex on the cell membrane, such as oxidized phospholipid [43,44,45], since H${}_{2}$O${}_{2}$ is added externally. Such oxidation-specific epitopes can be detected by pattern-recognition receptors on macrophages in the same way as the recognition of lipopolysaccharide (LPS) by toll-like receptor-4 (TLR4) [45], resulting in a significantly higher NO secretion level compared to that produced by vaccines made by PDT.

Regardless of using PDT or H${}_{2}$O${}_{2}$ as the source of oxidative stress, it seems that the nanovesicle vaccines generated by filter extrusion are roughly as effective as those produced by spontaneous release. The observation that the number of nanovesicles spontaneously released by LLC cells following PDT treatment rose in the first 6 h and then dropped to an equilibrium value (Figure 2c) could be ascribed to the reabsorption of nanovesicles by LLC cells to reach a dynamic equilibrium between continual ingestion and secretion of nanovesicles. As a result, the level of DAMP on nanovesicles collected at 24 h after oxidative stress could be as large as that produced by filter extrusion at 24 h. This is because the surface density of DAMP on nanovesicles is directly determined by that on the surfaces of stressed cells regardless of whether the nanovesicles are produced by direct outward budding or double invagination. These together with the similar numbers of nanovesicles per cell generated with these two methods (*ca.* 400 nanovesicles per cell) lead to similar efficacies of the vaccines made by the two different ways of nanovesicle production. Nevertheless, because the number of nanovesicles produced by spontaneous release may depend on the conditions of the cancer cells, whereas that produced by the filter extrusion does not, the latter could render a more reliable way for manufacturing vaccines. Moreover, since the two ways can be applied together to the same cell culture, by collecting the supernatant first after centrifugation and then re-suspending the cell pellet for filter extrusion, the two can be added together to double the number of vaccine nanovesicles for raising vaccine production throughput.

## 5. Conclusions

In this work, the efficacies of vaccines generated by applying different sources of oxidative stress on cancer cell cultures followed by spontaneous release or filter extrusion to produce cancer cell-derived DAMP-expressing nanovesicles on killing cancer cells in the co-culture of cancer cells and macrophages were studied. It seems that vaccines made by H${}_{2}$O${}_{2}$ kill LLC cells mainly through the activation of the cytotoxicity of macrophages toward LLC cells, while vaccines made by PDT kill LLC cells through both the uptake of vaccine nanovesicles by LLC cells and the activation of the cytotoxicity of macrophages. Although their dominant effecting pathways seem different, nanovesicle vaccines generated by H${}_{2}$O${}_{2}$ are as efficacious as those generated by PDT in diminishing cancer cell culture masses, providing a simpler way to manufacture vaccines. In addition, it was found that nanovesicle vaccines produced by filter extrusion are as effective as those produced by spontaneous release, rendering a more stable way for vaccine production. Since nanovesicle vaccines made by H${}_{2}$O${}_{2}$ and PDT kill cancer cells through different (main) routes and nanovesicles can be produced through spontaneous release and filter extrusion from the same cancer cell culture at the same time, the mixture of the four combinations may further provide a vaccine with higher efficiency and higher efficacy.

## Data Availability

Data are contained within the article.
